# Inhaled nitric oxide for acute respiratory distress syndrome in adults: a systematic review and meta-analysis

**DOI:** 10.1186/s40560-025-00845-4

**Published:** 2026-01-02

**Authors:** Yuta Nakamura, Yuki Kotani, Takatoshi Koroki, Hideki Tachibana, Shunta Tsutsumi, Toshiyuki Karumai, Yoshiro Hayashi

**Affiliations:** 1 Department of Intensive Care Medicine, Kameda Medical Center, 929 Higashi-cho, Kamogawa, Chiba 296-8602 Japan; 2 Emergency and Trauma Center, Kameda Medical Center, Kamogawa, Japan

**Keywords:** Systematic review, Meta-analysis, Critical care, Respiratory distress syndrome, Nitric oxide, Acute kidney injury, Extracorporeal membrane oxygenation

## Abstract

**Background:**

Although inhaled nitric oxide (iNO) is used as a rescue therapy in patients with acute respiratory distress syndrome (ARDS), its impact on patient-centered outcomes remains uncertain. To address this gap, we conducted a systematic review of randomized controlled trials (RCTs) to test the hypothesis that the addition of iNO to standard care improves survival in adult patients with ARDS.

**Methods:**

We searched PubMed, Embase, Cochrane Library, ClinicalTrials.gov, and WHO ICTRP for RCTs evaluating iNO in adult patients with ARDS through October 28, 2025. The primary outcome was mortality at the longest follow-up. Secondary outcomes included acute kidney injury (AKI), receipt of renal replacement therapy (RRT), duration of mechanical ventilation, length of intensive care unit stay, length of hospital stay, receipt of extracorporeal membrane oxygenation (ECMO), mean pulmonary artery pressure, partial pressure of arterial oxygen/fraction of inspiratory oxygen (PaO_2_/FiO_2_) ratio, elevated methemoglobin concentrations (> 5%), elevated nitrogen dioxide concentrations (> 3 ppm), extubation, and reintubation. We pooled data using a random-effects model, assessed the risk of bias with the Cochrane RoB 2 tool, and graded certainty with the GRADE approach.

**Results:**

We included 11 RCTs comprising 1302 patients. Only one study was of low risk of bias. iNO therapy may result in no difference in mortality at the longest follow-up (relative risk [RR], 1.07; 95% confidence interval [CI], 0.93–1.23; *I*^2^ = 0%; low certainty). iNO may improve PaO₂/FiO₂ ratio slightly (mean difference, 15.03 mmHg; 95% CI, 6.19–23.86; *I*^2^ = 0%; low certainty). The evidence is very uncertain about the effect on ECMO use (RR, 0.45; 95% CI, 0.10–2.17; *I*^2^ = 45%; very low certainty). iNO may increase the need for RRT (RR, 1.56; 95% CI, 1.17–2.08; *I*^2^ = 0%; low certainty). No clear differences were observed in other secondary outcomes. No study reported data on reintubation.

**Conclusions:**

Although iNO may improve oxygenation slightly, it may not confer survival or other patient-centered benefits and may increase the need for RRT. High-quality randomized evidence is needed to guide the optimal patient selection for this therapeutic option.

**Trial registration:**

PROSPERO (registration number: CRD42024573383).

**Supplementary Information:**

The online version contains supplementary material available at 10.1186/s40560-025-00845-4.

## Introduction

Acute respiratory distress syndrome (ARDS) is a life-threatening lung condition accounting for approximately 10% of intensive care unit (ICU) admissions, with a mortality rate near 40% [1]. Survivors frequently require prolonged mechanical ventilation and critical care and endure long-term physical, cognitive, and psychological sequelae [2–6]. Despite advances in supportive care, effective disease-modifying therapies remain limited [7, 8], underscoring the need for additional therapeutic options.

Inhaled nitric oxide (iNO) has been investigated as an adjunctive therapy for ARDS. By selectively dilating pulmonary vessels in well-ventilated lung regions, iNO enhances ventilation–perfusion matching, reduces intrapulmonary shunt, and augments oxygenation [9, 10]. Early small randomized controlled trials (RCTs) demonstrated physiologic benefits, including improved oxygenation and reduced mean pulmonary artery pressure [11, 12], but subsequent studies failed to show clinically meaningful outcome benefits [13]. A 2017 meta-analysis also raised concerns regarding potential nephrotoxicity [14]. Moreover, the 2021 Japanese ARDS guideline performed a systematic review of iNO therapy and suggested against its routine use [15]. However, the evidence base was derived from studies conducted more than two decades ago, many of which included pediatric populations, thereby limiting their relevance to contemporary adult practice.

The coronavirus disease 2019 (COVID-19) pandemic renewed interest in iNO. COVID-19 ARDS is characterized by profound hypoxemia with relatively preserved lung compliance, implicating prominent ventilation–perfusion mismatch and endothelial dysregulation [16]. These features provide a biological rationale for the use of pulmonary vasodilators. Current COVID-19 guidelines acknowledge iNO as a potential rescue option for refractory hypoxemia [17]. Importantly, recent RCTs conducted in patients with COVID-19 provide new data on both efficacy and safety. At the same time, concerns have grown regarding renal toxicity and the potential influence of iNO on extracorporeal membrane oxygenation (ECMO) use.

Despite uncertain clinical benefits, iNO remains widely used in real-world practice, particularly for severe hypoxemia and as an adjunct when considering ECMO [18, 19]. This persistent clinical use, emergence of new adult RCTs, and unresolved safety concerns highlight an important knowledge gap between historical evidence and modern clinical practice. Moreover, no recent systematic review has integrated COVID-19 and non-COVID-19 RCTs restricted to adults or evaluated key clinical outcomes, including mortality, kidney injury, and ECMO utilization, with prespecified subgroup analyses.

Given the biological and pharmacological plausibility, renewed use during the COVID-19 era, and availability of contemporary adult RCTs, a reassessment of iNO therapy in ARDS is warranted. We therefore conducted an updated systematic review and meta-analysis to evaluate the effect of iNO on clinically relevant outcomes in adult ARDS with prespecified subgroup analyses by COVID-19 status and ARDS severity.

## Methods

We followed the PRISMA 2020 statement [20] and prospectively registered the protocol with PROSPERO (registration number: CRD42024573383). The review question used a PICOS framework: In adult patients with ARDS defined by the author, does iNO plus usual care, compared with usual care alone, reduce mortality at the longest follow-up in RCTs?

### Search strategy and study selection

Two investigators (YN and YK) independently searched PubMed, EMBASE, the Cochrane Library, ClinicalTrials.gov, and WHO ICTRP from inception through October 28, 2025, without language restrictions. The complete search strategies are available in the Supplementary Material. We included RCTs comparing iNO plus usual care with usual care without iNO in adults with ARDS. Exclusions included pediatric studies (< 18 years), non-randomized trials, observational studies, reviews, commentaries/editorials, and studies not addressing the review question. Studies identified through clinical trial registries were included only if full-text publication was available, as the lack of full-text publication precludes adequate assessment of the risk of bias.

After de-duplication, four investigators (YN, TK, HT, and ST) independently screened titles/abstracts and assessed full texts; disagreements were resolved by discussion or adjudication by a third investigator (YK).

### Data collection

Two investigators (YN and ST) independently extracted data using a standardized form, with discrepancies resolved by consensus or adjudication by a third author (YK). Extracted variables included study characteristics, ARDS definitions, iNO dosing and duration, and outcomes [21]. The authors were contracted for clarification when necessary.

### Risk of bias and certainty of evidence

We assessed the risk of bias using the Cochrane Risk of Bias 2 (RoB 2) tool [22]. We graded the certainty of the evidence with the GRADE approach and generated Summary of Findings tables in GRADEpro software [23]. Publication bias for the primary outcome was evaluated by visual inspection of the funnel plot.

### Outcomes

The primary outcome was mortality at the longest follow-up. Secondary outcomes included acute kidney injury (AKI), renal replacement therapy (RRT), duration of mechanical ventilation, ICU and hospital length of stay, ECMO, mean pulmonary artery pressure (mPAP), PaO_2_/FiO_2_ ratio, elevated methemoglobin > 5%, nitrogen dioxide > 3 ppm, extubation, and reintubation. Thresholds for methemoglobinemia and nitrogen dioxide were aligned with prior RCTs and systematic reviews [14]. All these outcome data were collected at the longest follow-up available. AKI definitions followed each trial. Outcome selection was guided by the core outcome set for ventilation trials in critically ill populations [24].

### Statistical analysis

We pooled dichotomous outcomes as relative risk (RR) using Mantel–Haenszel random-effects models and continuous outcomes as mean differences (MD) using inverse-variance random-effects models, each with 95% confidence intervals (CIs). Between-study heterogeneity was quantified with Tau^2^ and *I*^2^. Two-sided *P* < 0.05 was considered statistically significant.

Prespecified subgroup analysis examined COVID-19 versus non-COVID-19 etiology and was applied to the primary and all secondary outcomes. Analyses were conducted in RevMan Web (v8.11.0) [25].

### Protocol deviations

After registering the protocol in PROSPERO (CRD42024573383) on August 5, 2024, we made several modifications to the review protocol. First, we removed health-related quality of life as an outcome due to the lack of adequate rationale between iNO therapy and this outcome measure. Second, we conducted a post hoc subgroup analysis based on ARDS severity (moderate-to-severe ARDS [baseline PaO_2_/FiO_2_ < 200] vs. any ARDS severity) to explore whether baseline severity could modify the treatment effects of iNO therapy. Third, we initially planned to perform a sensitivity analysis restricted to studies at low risk of bias. However, due to the scarcity of such studies, we instead performed a sensitivity analysis excluding high risk of bias studies. Both the subgroup and sensitivity analyses were applied across all outcomes. Fourth, to assess the robustness of the conventional meta-analytic findings, we performed a trial sequential analysis for the primary outcome, calculating the diversity-adjusted information size under assumptions of a two-sided alpha = 0.05, power = 0.8, and a 10% relative risk reduction; the baseline risk was the average mortality in the control groups [26]. Analyses were performed using TSA Viewer (version 0.9.5.10 Beta, Copenhagen Trial Unit, Centre for Clinical Intervention Research, Rigshospitalet, Copenhagen, Denmark).

## Results

**Fig. 1:**
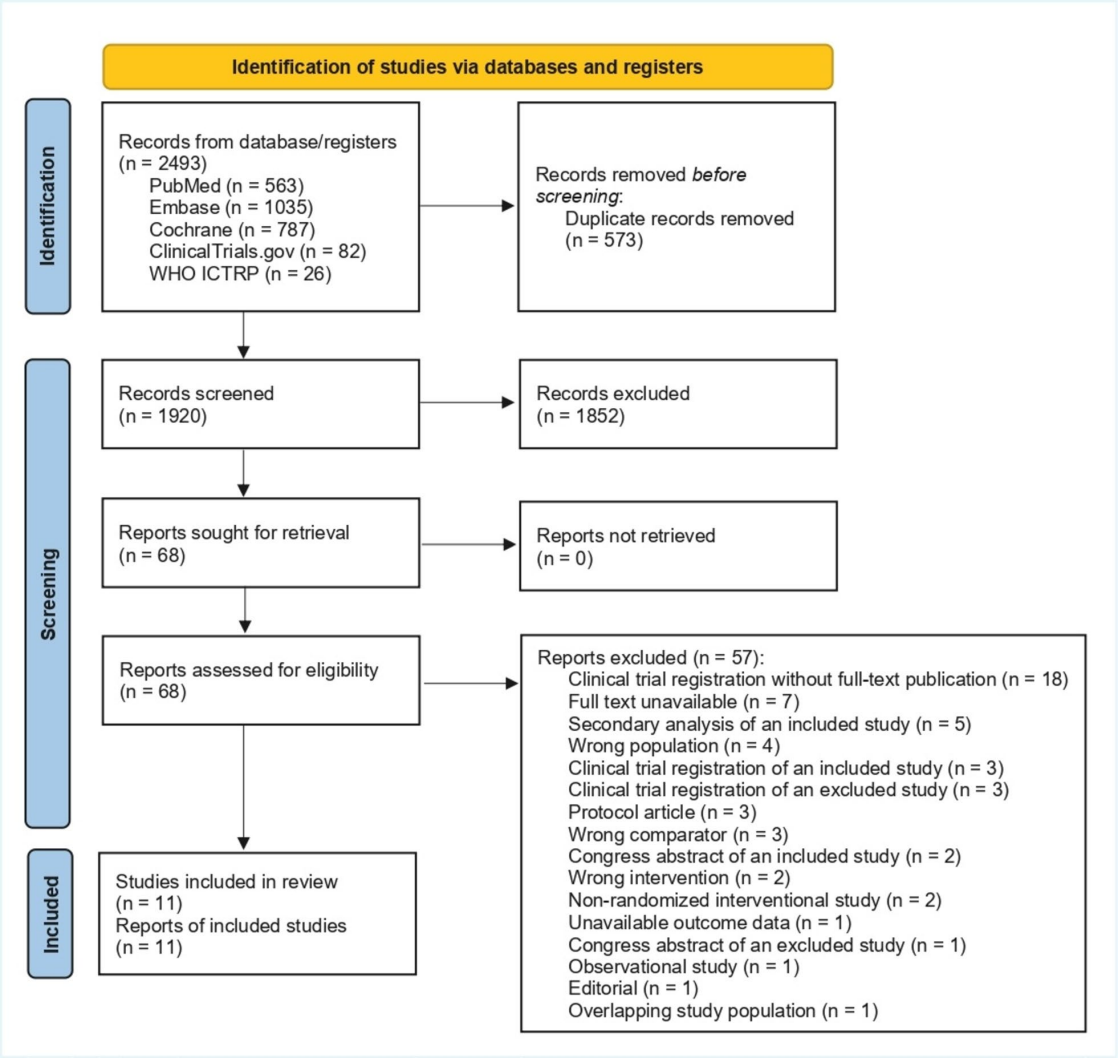
PRISMA 2020 flow diagram. Process of identification, screening, eligibility assessment, and inclusion of randomized trials evaluating inhaled nitric oxide in adult ARDS

We identified 2493 records through database and registry searches. After removal of 573 duplicates, 1920 records underwent title/abstract screening, of which 1852 were excluded. We assessed 68 full-text reports for eligibility; 57 were excluded. Ultimately, 11 RCTs (*n* = 1302) met the eligibility criteria [11–13, 27–34] (Fig. 1). Study characteristics are summarized in Table 1. Major exclusions are detailed in Supplementary Table S1. Trials were published between 1998 and 2023, and six were multicenter [11, 13, 27, 28, 32, 33]. In one mixed adult-pediatric trial, four pediatric participants were excluded from our analyses [29]. Initial iNO doses ranged from 2.5 to 80 ppm. The risk of bias was judged to be low in one study, of some concern in six, and high in four (Supplementary Table S2).

**Table 1:** Characteristics of the included studies

First author, publication year	Setting	No. of centers	No. of patients	Initial iNO dose	Control	Timing of mortality assessment
Fenza R, 2023	Adults, mechanically ventilated COVID-19	5	193	80 ppm	Usual care	90 days
Dellinger RP, 1998	Adults, ARDS	30	177	Up to 80 ppm	Placebo	28 days
Lundin S, 1999	Adults, ARDS	43	180	Up to 40 ppm	Usual care	90 days
Taylor RW, 2004	Adults, ARDS	46	385	5 ppm	Placebo	1 year
Michael JR, 1998	Adults and children, ARDS	1	36	5 ppm	Usual care	Not reported
Troncy E, 1998	Adults, ARDS*	1	30	2.5 ppm	Usual care	30 days
Merlin M, 2022	Adults, moderate-to-severe COVID-19	1	25	10 ppm	Usual care	28 days
Gerlach H, 2003	Adults, ARDS	1	40	10 ppm	Usual care	Not specified
Payen D, 1999	> 15 years, Murray lung injury score: 2–3	23	203	10 ppm	Placebo	90 days
Mehta S, 2001	Adults, ARDS	1	14	5 ppm	Usual care	Not specified
Schwebel C, 1997	Adults, ARDS	17	19	10 ppm	Placebo	Not specified

*iNO* inhaled nitric oxide; *ARDS* Acute respiratory distress syndrome. * Murray lung injury score ≥ 2.5

### Primary outcome

**Fig. 2:**
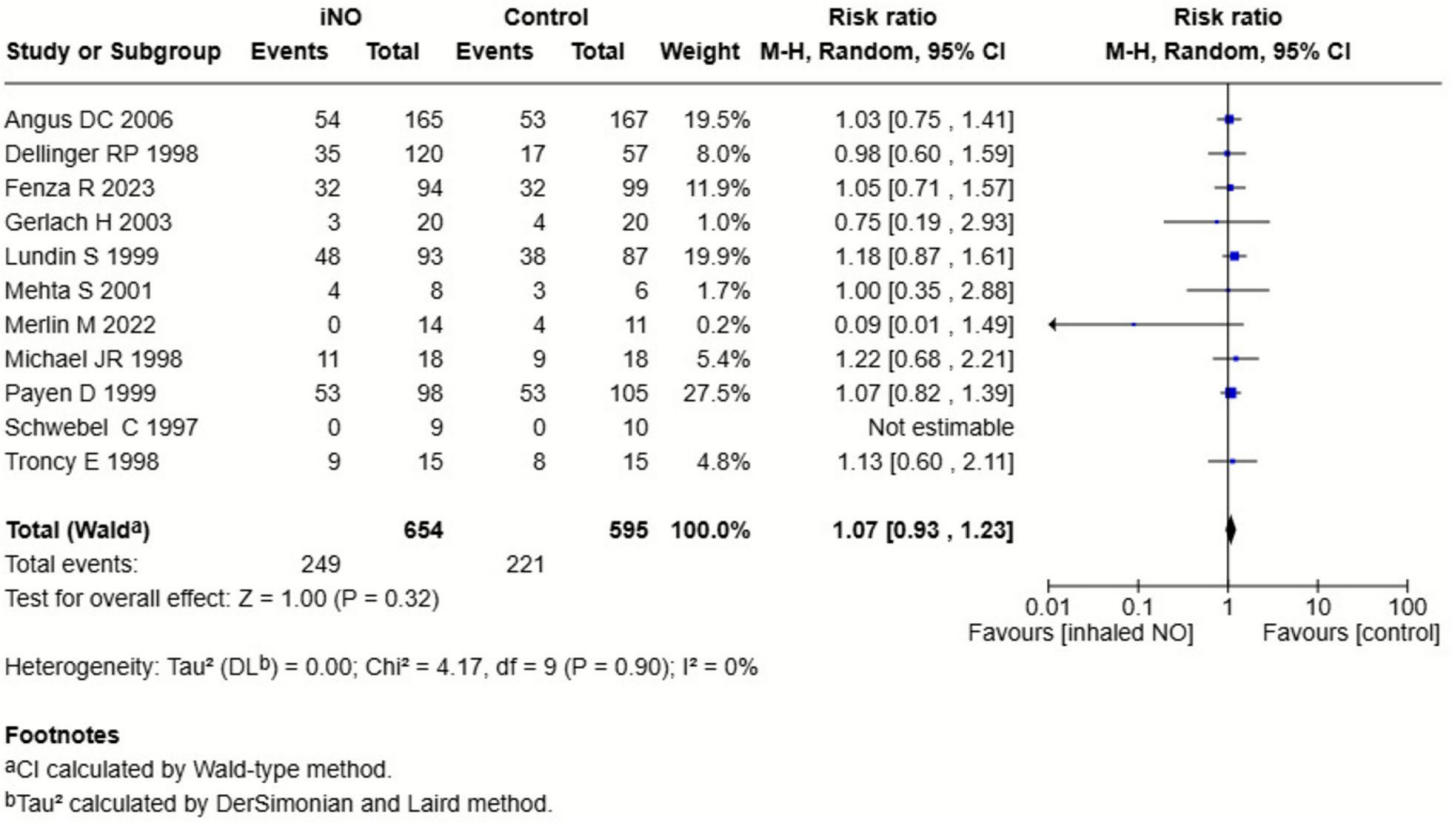
Forest plot of all-cause mortality at longest follow-up. Relative risks with 95% confidence intervals from included randomized controlled trials. Inhaled nitric oxide showed no significant effect compared with control (RR 1.07, 95% CI 0.93–1.23; *I*^2^ = 0%)

**Fig. 3:**
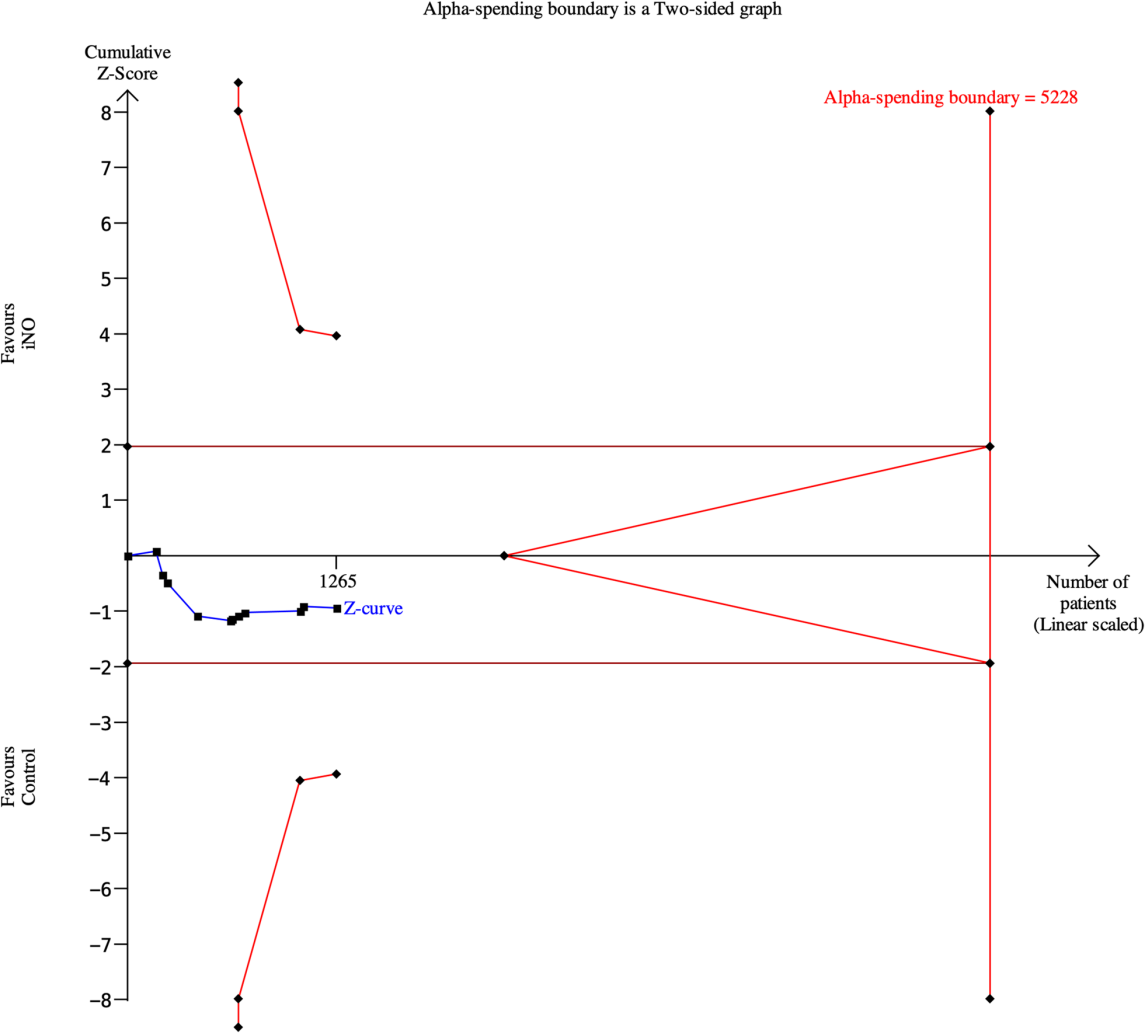
Trial sequential analysis of mortality. Cumulative evidence did not cross monitoring boundaries for benefit, harm, or futility. Required information size was not reached, indicating insufficient evidence for firm conclusions

The pooled analyses indicated that iNO may result in no difference in mortality at the longest follow-up (RR, 1.07; 95% CI, 0.93–1.23; *P* = 0.32; *I*^2^ = 0%; low certainty; Table 2 and Fig. 2). Visual inspection of the funnel plot suggested no publication bias (Supplementary Figure S1). Treatment effects did not differ by ARDS etiology (COVID-19 vs. non-COVID-19) or by severity restricted to moderate-to-severe ARDS (Supplementary Figures S2, S3). A sensitivity analysis excluding trials judged to have an overall high risk of bias yielded a similar estimate (Supplementary Figure S4). Trial sequential analysis indicated that the required information size (*n* = 5228) was not reached, signifying insufficient evidence to draw a definitive conclusion regarding a mortality effect of iNO (Fig. 3). The timing of mortality assessment is summarized in Table 1.

**Table 2:** Effects of inhaled nitric oxide for ARDS on the primary outcome

Outcome	No. of studies	Inhaled nitric oxide	Control	Risk ratio/mean difference (95% CI)	P value	*I*^2^	P for interaction
Primary outcome							
Mortality at the longest follow-up	11	249/654 (38%)	221/595 (37%)	1.07 (0.93–1.23)	0.32	0%	
*ARDS etiology*							0.47
COVID-19	2	32/108 (30%)	36/110 (33%)	0.45 (0.04–4.83)	0.51	67%	
Non-COVID-19	9	217/546 (40%)	185/485 (38%)	1.08 (0.93–1.25)	0.29	0%	
*Severity of ARDS*							0.69
Moderate-to-severe ARDS	7	154/366 (42%)	124/303 (41%)	1.10 (0.92–1.30)	0.30	0%	
Any severity ARDS	4	95/288 (33%)	97/292 (33%)	1.03 (0.82–1.31)	0.72	2%	
Excluding high risk-of-bias studies	7	183/524 (35%)	157/459 (34%)	1.07 (0.90–1.27)	0.42	0%	

*CI* Confidence interval; *ARDS* Acute respiratory distress syndrome; *COVID-19* coronavirus disease 2019

### Secondary outcomes

The results of the secondary outcomes are summarized in Table 3.

**Table 3:** Effects of inhaled nitric oxide for ARDS on the secondary outcomes

Outcome	No. of studies	Inhaled nitric oxide	Control	Risk ratio/mean difference (95% CI)	P value	*I*^2^
Secondary outcomes						
Acute kidney injury	5	158/597 (27%)	122/541 (23%)	1.34 (0.95–1.89)	0.10	58%
Renal replacement therapy	3	89/285 (31%)	58/291 (20%)	1.56 (1.17–2.08)	0.002	0%
Duration of mechanical ventilation, day	4			2.24 (− 3.64–8.11)	0.46	82%
ICU length of stay, day	3			− 0.20 (− 2.69–2.28)	0.87	0%
Hospital length of stay, day	2			− 0.67 (− 4.47–3.13)	0.73	0%
Receipt of ECMO	2	5/114 (4.4%)	11/119 (9.2%)	0.45 (0.10–2.17)	0.32	45%
Mean pulmonary artery pressure, mmHg	4			− 2.44 (− 4.63– − 0.26)	0.03	13%
PaO_2_/FiO_2_ ratio, mmHg	8			15.03 (6.19–23.86)	0.0009	0%
Extubation	1	6/15 (40%)	5/15 (33%)	1.20 (0.47–3.09)	0.38	NA
Methemoglobin concentrations > 5%	10	4/587 (0.7%)	3/522 (0.6%)	0.90 (0.20–4.14)	0.89	0%
Nitrogen dioxide concentrations > 3 ppm	6	3/453 (0.7%)	0/396 (0%)	3.36 (0.18–63.89)	0.42	NA

*CI* Confidence interval; *ICU* Intensive care unit; *ECMO* Extracorporeal membrane oxygenation; *PaO*_*2*_ Partial pressure of arterial oxygen; *FiO*_*2*_ Fraction of inspired oxygen

### Oxygenation and pulmonary artery pressure

iNO therapy may slightly increase PaO₂/FiO₂ (MD, 15.03 mmHg; 95% CI, 6.19–23.86; *P* = 0.0009; *I*^2^ = 0%; low certainty). Evidence for an effect on mPAP was very uncertain (MD, − 2.48 mmHg; 95% CI, − 5.48–0.52; *P* = 0.11; *I*^2^ = 42%; very low certainty).

### Renal outcomes

The effect on AKI was very uncertain (RR, 1.34; 95% CI, 0.95–1.89; *P* = 0.10; *I*^2^ = 58%; very low certainty). iNO may increase the need for RRT (RR, 1.56; 95% CI, 1.17–2.08; *P* = 0.002; *I*^2^ = 0%; low certainty).

### ECMO and duration of mechanical ventilation

The evidence was very uncertain for the effects of iNO on ECMO use (RR, 0.45; 95% CI, 0.10–2.17; *P* = 0.32; *I*^2^ = 45%; very low certainty) and on duration of mechanical ventilation that was detected (MD, 2.24 days; 95% CI, − 3.64–8.11; *P* = 0.46; *I*^2^ = 82%; very low certainty).

### ICU and hospital length of stay

iNO therapy may result in no difference in ICU length of stay (MD, 1.38 days; 95% CI, − 1.99–4.76; *P* = 0.87; *I*^2^ = 0%; low certainty) or in hospital length of stay (MD, − 0.67 days; 95% CI, − 4.47–3.13; *P* = 0.73; *I*^2^ = 0%; low certainty).

### Extubation

Evidence for an effect on extubation rate was very uncertain (RR, 1.20; 95% CI, 0.47–3.09; *P* = 0.71; very low certainty).

### Methemoglobin and nitrogen dioxide concentrations

Evidence was very uncertain regarding differences in the proportion of patients with methemoglobin > 5% (RR, 0.90; 95% CI, 0.80–4.14; *P* = 0.89; *I*^2^ = 0%; very low certainty) and with nitrogen dioxide concentrations > 3 ppm (RR, 3.36; 95% CI, 0.18–63.89; *P* = 0.42; very low certainty).

### Reintubation

No study reported reintubation rates.

### Subgroup analyses

#### COVID-19 versus non-COVID-19 ARDS

A significant interaction for AKI was observed (P for interaction = 0.01), with no effects in COVID-19 ARDS (RR, 0.99; 95% CI, 0.82–1.20; *P* = 0.93) and increased AKI risk in non-COVID-19 ARDS (RR, 1.55; 95% CI, 1.14–2.10; *I*^2^ = 0%). No heterogeneity of treatment effects was detected for other secondary outcomes (Supplementary Table S4).

#### Moderate-to-severe ARDS versus any ARDS severity

AKI demonstrated significant effect modification (*P* for interaction = 0.02). AKI risk increased with iNO in moderate-to-severe ARDS (RR, 1.55; 95% CI, 1.12–2.15; *I*^2^ = 0%) but showed no difference when trials enrolled patients of any severity (RR, 1.01; 95% CI, 0.84–1.21; *I*^2^ = 0%). A further effect modification was observed for duration of mechanical ventilation (P for interaction = 0.03), with point estimates favoring control in moderate-to-severe ARDS (MD, 6.05 days; 95% CI, − 0.72–12.83; *I*^2^ = 58%) and favoring iNO when any severity was included (MD, − 1.72 days; 95% CI, − 3.47–0.03; *I*^2^ = 0%). Other outcomes showed no evidence of effect modification by severity (Supplementary Table S5).

#### Sensitivity analysis

Excluding trials at high risk of bias did not materially alter the secondary-outcome estimates, including the need for renal replacement therapy (RR, 1.74; 95% CI, 1.19–2.55; *P* = 0.004) and PaO_2_/FiO_2_ ratio (MD, 14.17 mmHg; 95% CI, 4.71–23.62; *P* = 0.003) (Supplementary Table S6).

## Discussion

### Key findings

Across 11 RCTs including 1302 adults with ARDS, iNO may slightly improve oxygenation but may not improve mortality or other patient-centered outcomes and may increase the need for RRT.

### Comparison with previous literature

Earlier systematic reviews often combined pediatric and adult populations, mixed diverse clinical indications (e.g., cardiac surgery, organ transplant, sepsis), and used heterogenous comparators such as recruitment maneuvers instead of usual care or placebo. In addition, previous reviews mostly included studies published more than 10 years ago and predated the COVID-19 pandemic [9, 14, 35–37]. These factors limit their applicability to contemporary adult ARDS. By focusing on adult RCTs, limiting comparators to usual care or placebo, and incorporating recent trials, our analysis provides an updated estimate of the effects of iNO.

Outcome selection also varied. Previous reviews synthesized physiological and clinical endpoints [9, 14, 37], while our review emphasized patient-centered and organ support-related outcomes reflecting current practice (use of RRT and ECMO). Despite these methodological differences, several conclusions were consistent, including improvements in oxygenation, rare elevations in methemoglobin and nitrogen dioxide levels, and the lack of a survival benefit.

Moreover, differences in statistical methods may also contribute to divergent interpretations. Previous reviews performed subgroup analysis based on etiology or age [9, 14], while our meta-analysis conducted a prespecified subgroup analysis of COVID-19 versus non-COVID-19 ARDS and performed a trial sequential analysis on mortality. These differences help contextualize our findings within the existing literature.

Mortality reduction was not demonstrated, and the confidence interval encompassed potential harm. The difference from prior neutral meta-analyses [9, 37] may be due to the exclusion of pediatric trials and the inclusion of newer studies. Our observation that iNO may increase RRT use aligns with previous reports showing increased renal risks, although previous works did not specifically collect the need for RRT [9, 14, 35, 36].

A major concern is the heterogeneity between older non-COVID-19 ARDS trials and recent COVID-19 studies. While clinical and pathophysiological contexts differ substantially, our subgroup analyses (COVID-19 vs. non-COVID-19, moderate-to-severe ARDS vs. any severity) help delineate potential effect modification. Although combining such data mathematically has limitations, in the absence of adequately powered recent RCTs, a comprehensive synthesis remains informative for clinicians and guideline developers.

Importantly, the renal signal might differ by etiology: iNO may increase acute kidney injury in non-COVID-19 ARDS, whereas it may have no effect in COVID-19 ARDS. This might highlight the need for cautious, selective use of iNO rather than routine application.

In contrast, perioperative iNO during cardiac surgery has been linked to reduced kidney injury [35, 38, 39], a difference likely driven by mechanisms specific to the surgical setting (e.g., cardiopulmonary bypass-related hemolysis and NO depletion, right ventricular unloading, and renal microcirculation changes) that are less relevant to ARDS [38].

The presence or absence of COVID-19 as the underlying etiology of ARDS may modulate the renal effects of iNO. The prespecified subgroup analysis showed a very uncertain effect of iNO on AKI in COVID-19 ARDS. Mechanistically, systemic inflammation in ARDS creates a high baseline NO burden [40]. By contrast, COVID-19 ARDS is characterized by NO depletion [16] and microthrombosis [41], conditions in which iNO may mitigate renal microcirculatory damage [16]. However, these observations remain speculative, and whether iNO confers a renoprotective effect in COVID-19 remains to be determined.

The observed signal regarding ECMO use may suggest a potential role for iNO in severe ARDS. Only two studies reported ECMO utilization; although the pooled point estimate favored iNO, the confidence interval crossed the null. This pattern is consistent with prior pediatric reviews reporting fewer ECMO runs with iNO [42, 43] and with a contemporary COVID‑19 ARDS cohort in which nearly half of the patients meeting ECMO criteria (PaO₂/FiO₂ < 80) achieved adequate oxygenation within six hours of iNO therapy and no longer met ECMO thresholds [44]. Taken together, these findings suggest that iNO may provide short-term oxygenation support, function as a bridge to ECMO, and, when extracorporeal circuits are scarce or ECMO initiation is delayed, serve as a means to avert ECMO in selected patients. However, due to the small evidence base and the resulting imprecision (especially about ECMO use), the study findings should be interpreted as hypothesis-generating. To evaluate such hypothetical clinical roles of iNO therapy, confirmation will require adequately powered and methodologically rigorous RCTs.

### Implications for clinical practice and future research

Given the lack of consistent benefits in patient-centered outcomes and the low to very low certainty of evidence in the majority of the outcomes, our findings support a conditional recommendation against the routine use of iNO therapy in adults with ARDS. Clinical application should be considered only in carefully selected patients most likely to benefit, with heightened caution in those with renal impairment, given the observed increase in RRT use. Several considerations should inform future studies. First, given the resource-intensive nature of ECMO, further evaluation is needed to determine whether iNO can reduce the need for ECMO initiation in severe ARDS. Second, mechanistic studies should investigate the relationship between exogenous iNO administration and worsening renal dysfunction to explain the contrasting renal effects observed in ARDS versus cardiac surgery populations.

### Strengths and limitations

This review represents the most contemporary assessment of iNO therapy in adults with ARDS, incorporating trials conducted during the COVID-19 era. Prespecified subgroup analyses by ARDS etiology and severity help delineate potential heterogeneity of treatment effects. Restricting inclusion to RCTs minimizes bias and strengthens the credibility of the findings.

Several limitations merit consideration. First, only one study was judged to be at low risk of bias; however, sensitivity analyses excluding trials at high risk of bias yielded similar results, supporting the robustness of the main findings. Second, the included studies exhibited substantial clinical heterogeneity, particularly with respect to ARDS severity. The clinical phenotype of COVID-19 ARDS differs from that of classic ARDS [16]. We addressed these issues by conducting subgroup analyses by etiology and severity, which suggested potentially different effects across outcomes. Third, the trials span more than 25 years, during which the standard of care for ARDS has evolved (e.g., the widespread adoption of prone positioning and lung-protective ventilation). Fourth, the inconsistent findings between AKI and RRT might have derived from between-study variations in AKI diagnostic criteria and RRT initiation criteria. Notably, none of the three studies reporting RRT results explicitly defined the initiation criteria [27, 28, 32]. Fifth, the subgroup analyses based on the etiology and severity of ARDS should be interpreted as hypothesis-generating given a limited number of studies and events.

## Conclusions

In adults with ARDS, iNO may slightly improve oxygenation without improving mortality or other patient-centered outcomes and may increase the need for RRT. Routine use of iNO in adult ARDS cannot be recommended, given the lack of survival benefit and the increased need for renal replacement therapy. However, potential niche roles—such as temporary oxygenation support, bridging to ECMO, or selective use in COVID-19 ARDS—warrant cautious exploration. Further research should be hypothesis-driven and focused on clearly defined patient subsets rather than large confirmatory trials.

## Supplementary Information

Additional file 1.

## Data Availability

We collected the summary data from published randomized trials. All the data generated or analyzed for this study are included in this published article and its supplementary file. Further information is available from the corresponding authors upon reasonable request.

## Abbreviations

PEEP: Positive end-expiratory pressure
ICU: Intensive care unit
CI: Confidence interval
GRADE: Grading of Recommendations Assessment, Development and Evaluation
PRISMA: Preferred Reporting Items for Systematic Reviews and Meta-Analyses
RR: Risk ratios
iNO: Inhaled nitric oxide
