# Supplementary material for: Inhaled nitric oxide for acute respiratory distress syndrome in adults: a systematic review and meta-analysis
Source: J Intensive Care. 2026 Jan 2;14:10. doi: 10.1186/s40560-025-00845-4 (PMC12866168; doi:10.1186/s40560-025-00845-4)
Supplement: Supplementary file 1 — Additional file 1. [file 40560_2025_845_MOESM1_ESM.docx]

**Supplementary material**

**Inhaled nitric oxide therapy for acute respiratory distress syndrome: a meta-analysis of randomized controlled trials**

**Authors**

Yuta Nakamura, Yuki Kotani, Takatoshi Koroki, Hideki Tachibana, Shunta Tsutsumi, Toshiyuki Karumai, Yoshiro Hayashi

**Table of contents**

Search strategy for systematic literature review 3

PRISMA 2020 checklist 5

Fig. S1. Funnel plot for mortality at the longest follow-up available 8

Fig. S2. Forest plot of subgroup analysis by ARDS etiology for mortality at the longest follow-up 9

Fig. S3. Forest plot of subgroup analysis by ARDS severity for mortality at the longest follow-up 10

Fig. S4. Forest plot for mortality at the longest follow-up in studies without high risk of bias 11

Table S1. Major exclusions and reasons for exclusion, in order of year of publication12

Table S2. Risk of bias assessment of the included studies 14

Table S3. GRADE evaluation 15

Table S4. Subgroup analyses of secondary outcomes comparing COVID-19 and non-COVID-19 18

Table S5. Subgroup analyses of secondary outcomes based on ARDS severity 19

Table S6. Sensitivity analysis for secondary outcomes without high risk of bias 21

Supplementary references 22

# Search strategy for systematic literature review

PubMed

#1 Anoxia[MeSH Terms] OR Anoxemia[MeSH Terms] OR "Respiratory Paralysis"[MeSH Terms] OR "Respiratory Insufficiency"[MeSH Terms] OR "Respiratory Distress Syndrome, Newborn"[MeSH Terms] OR Respiratory Distress Syndrome, Adult[MeSH Terms] OR Acute[Title/Abstract] AND (hypox*[Title/Abstract] OR respiratory[Title/Abstract]) OR respirat*[Title/Abstract] AND (distress[Title/Abstract] OR failure[Title/Abstract]) OR "lung injury"[Title/Abstract] OR (hypoxia[Title/Abstract] OR hypoxemia[Title/Abstract] OR AHRF[Title/Abstract] OR ARDS[Title/Abstract] OR ALI[Title/Abstract]) OR COVID-19[Title/Abstract]

#2 "Nitric Oxide"[MeSH Terms] OR "Endothelium-Dependent Relaxing Factors"[MeSH Terms] OR (Nitric[Title/Abstract] AND oxide[Title/Abstract])

#3 ((randomized controlled trial[Publication Type] OR controlled clinical trial[Publication Type] OR randomized[Title/Abstract] OR placebo[Title/Abstract] OR "clinical trials as topic"[MeSH Terms] OR randomly[Title/Abstract] OR trial[Title]) AND humans[MeSH Terms])

#4 #1 AND #2 AND #3

Cochrane Library

#1 MeSH descriptor Anoxia explode all trees

#2 MeSH descriptor Respiratory Paralysis explode all trees

#3 MeSH descriptor Respiratory Insufficiency explode all trees

#4 MeSH descriptor Respiratory Distress Syndrome explode all trees

#5 (Acute near (hypox* or respiratory)):ti,ab

#6 (respirat* near (distress or failure)):ti,ab

#7 lung injury

#8 (hypoxia or hypoxemia):ti

#9 AHRF or ARDS or ALI

#10 COVID-19

#11 (#1 OR #2 OR #3 OR # OR #5 OR #6 OR #7 OR #8 OR #9 OR #10)

#12 MeSH descriptor Nitric Oxide explode all trees

#13 MeSH descriptor Endothelium‐Dependent Relaxing Factors explode all trees

#14 Nitric near oxide

#15 (#12 OR #13 OR #14)

#16 (#11 AND #15)

Embase

#1 'anoxia'/exp OR 'anoxemia'/exp

#2 'respiratory paralysis'/exp

#3 'respiratory insufficiency'/exp

#4 'adult respiratory distress syndrome'/exp

#5 'newborn respiratory distress syndrome'/exp

#6 (acute NEAR/3 (hypox* OR respiratory)):ti,ab

#7 (respirat* NEAR/3 (distress OR failure)):ti,ab

#8 lung injury:ti,ab

#9 (hypoxia OR hypoxemia):ti,ab

#10 AHRF:ti,ab OR ARDS:ti,ab OR ALI:ti,ab

#11 COVID-19:ti,ab

#12 #1 OR #2 OR #3 OR #4 OR #5 OR #6 OR #7 OR #8 OR #9 OR #10 OR #11

#13 'nitric oxide'/exp

#14 'endothelium dependent relaxing factor'/exp

#15 (nitric NEAR/3 oxide):ti,ab

#16 #13 OR #14 OR #15

#17 'random*':ti,ab,kw OR 'randomized controlled trial':it OR 'controlled clinical trial':it OR 'randomized controlled trial (topic)'/exp OR 'randomization'/exp

#18 'review':ti OR 'meta*':ti OR 'animal':ti,ab,kw OR 'mouse':ti,ab,kw OR 'mice':ti,ab,kw OR 'protocol':ti OR 'murine':ti,ab,kw OR 'newborn':ti,ab,kw OR 'pediatric':ti,ab,kw OR 'neonatal':ti,ab,kw OR 'survey':ti OR 'cohort study':ti OR 'cohort analysis':ti OR 'retrospective study':ti OR 'study protocol':ti OR 'case-control':ti OR 'rabbit':ti OR 'squirrel':ti OR 'pig':ti OR 'rat':ti OR 'cats':ti,ab,kw OR 'animal*':kw OR 'animal*':ti OR 'rats':ti OR 'cross-sectional study':ti,ab,kw OR 'comment':it OR 'editorial':it

#19 #12 AND #16 AND #17 NOT #18

# PRISMA 2020 checklist

| **Section and Topic** | **Item #** | **Checklist item** | **Location where item is reported** |
| --- | --- | --- | --- |
| **TITLE** | | |  |
| Title | 1 | Identify the report as a systematic review. | 1 |
| **ABSTRACT** | | |  |
| Abstract | 2 | See the PRISMA 2020 for Abstracts checklist. | 2-3 |
| **INTRODUCTION** | | |  |
| Rationale | 3 | Describe the rationale for the review in the context of existing knowledge. | 4-5 |
| Objectives | 4 | Provide an explicit statement of the objective(s) or question(s) the review addresses. | 5 |
| **METHODS** | | |  |
| Eligibility criteria | 5 | Specify the inclusion and exclusion criteria for the review and how studies were grouped for the syntheses. | 5 |
| Information sources | 6 | Specify all databases, registers, websites, organisations, reference lists and other sources searched or consulted to identify studies. Specify the date when each source was last searched or consulted. | 5 |
| Search strategy | 7 | Present the full search strategies for all databases, registers and websites, including any filters and limits used. | Supplemental Material |
| Selection process | 8 | Specify the methods used to decide whether a study met the inclusion criteria of the review, including how many reviewers screened each record and each report retrieved, whether they worked independently, and if applicable, details of automation tools used in the process. | 5-6 |
| Data collection process | 9 | Specify the methods used to collect data from reports, including how many reviewers collected data from each report, whether they worked independently, any processes for obtaining or confirming data from study investigators, and if applicable, details of automation tools used in the process. | 6 |
| Data items | 10a | List and define all outcomes for which data were sought. Specify whether all results that were compatible with each outcome domain in each study were sought (e.g. for all measures, time points, analyses), and if not, the methods used to decide which results to collect. | 6-7 |
|  | 10b | List and define all other variables for which data were sought (e.g. participant and intervention characteristics, funding sources). Describe any assumptions made about any missing or unclear information. | 7 |
| Study risk of bias assessment | 11 | Specify the methods used to assess risk of bias in the included studies, including details of the tool(s) used, how many reviewers assessed each study and whether they worked independently, and if applicable, details of automation tools used in the process. | 6 |
| Effect measures | 12 | Specify for each outcome the effect measure(s) (e.g. risk ratio, mean difference) used in the synthesis or presentation of results. | 7 |
| Synthesis methods | 13a | Describe the processes used to decide which studies were eligible for each synthesis (e.g. tabulating the study intervention characteristics and comparing against the planned groups for each synthesis (item #5)). | 7 |
|  | 13b | Describe any methods required to prepare the data for presentation or synthesis, such as handling of missing summary statistics, or data conversions. | 7 |
|  | 13c | Describe any methods used to tabulate or visually display results of individual studies and syntheses. | 7 |
|  | 13d | Describe any methods used to synthesize results and provide a rationale for the choice(s). If meta-analysis was performed, describe the model(s), method(s) to identify the presence and extent of statistical heterogeneity, and software package(s) used. | 7 |
|  | 13e | Describe any methods used to explore possible causes of heterogeneity among study results (e.g. subgroup analysis, meta-regression). | 7 |
|  | 13f | Describe any sensitivity analyses conducted to assess robustness of the synthesized results. | 7 |
| Reporting bias assessment | 14 | Describe any methods used to assess risk of bias due to missing results in a synthesis (arising from reporting biases). | 7 |
| Certainty assessment | 15 | Describe any methods used to assess certainty (or confidence) in the body of evidence for an outcome. | 7 |
| **RESULTS** | | |  |
| Study selection | 16a | Describe the results of the search and selection process, from the number of records identified in the search to the number of studies included in the review, ideally using a flow diagram. | 8, Fig. 1 |
|  | 16b | Cite studies that might appear to meet the inclusion criteria, but which were excluded, and explain why they were excluded. | 8 |
| Study characteristics | 17 | Cite each included study and present its characteristics. | 8 |
| Risk of bias in studies | 18 | Present assessments of risk of bias for each included study. | 8, Supplemental material |
| Results of individual studies | 19 | For all outcomes, present, for each study: (a) summary statistics for each group (where appropriate) and (b) an effect estimate and its precision (e.g. confidence/credible interval), ideally using structured tables or plots. | 8-10, Table 1 |
| Results of syntheses | 20a | For each synthesis, briefly summarise the characteristics and risk of bias among contributing studies. | 8, Table 2, Supplemental material |
|  | 20b | Present results of all statistical syntheses conducted. If meta-analysis was done, present for each the summary estimate and its precision (e.g. confidence/credible interval) and measures of statistical heterogeneity. If comparing groups, describe the direction of the effect. | 8-11, Table 2 |
|  | 20c | Present results of all investigations of possible causes of heterogeneity among study results. | 10-11, Table 2 |
|  | 20d | Present results of all sensitivity analyses conducted to assess the robustness of the synthesized results. | 8-11, Supplemental material |
| Reporting biases | 21 | Present assessments of risk of bias due to missing results (arising from reporting biases) for each synthesis assessed. | Supplemental material |
| Certainty of evidence | 22 | Present assessments of certainty (or confidence) in the body of evidence for each outcome assessed. | Supplemental material |
| **DISCUSSION** | | |  |
| Discussion | 23a | Provide a general interpretation of the results in the context of other evidence. | 11-13 |
|  | 23b | Discuss any limitations of the evidence included in the review. | 13-14 |
|  | 23c | Discuss any limitations of the review processes used. | 14 |
|  | 23d | Discuss implications of the results for practice, policy, and future research. | 13 |
| **OTHER INFORMATION** | | |  |
| Registration and protocol | 24a | Provide registration information for the review, including register name and registration number, or state that the review was not registered. | 5 |
|  | 24b | Indicate where the review protocol can be accessed, or state that a protocol was not prepared. | 5 |
|  | 24c | Describe and explain any amendments to information provided at registration or in the protocol. | Not applicable |
| Support | 25 | Describe sources of financial or non-financial support for the review, and the role of the funders or sponsors in the review. | 15 |
| Competing interests | 26 | Declare any competing interests of review authors. | 15 |
| Availability of data, code and other materials | 27 | Report which of the following are publicly available and where they can be found: template data collection forms; data extracted from included studies; data used for all analyses; analytic code; any other materials used in the review. | 15 |

## Fig. S1. Funnel plot for mortality at the longest follow-up available


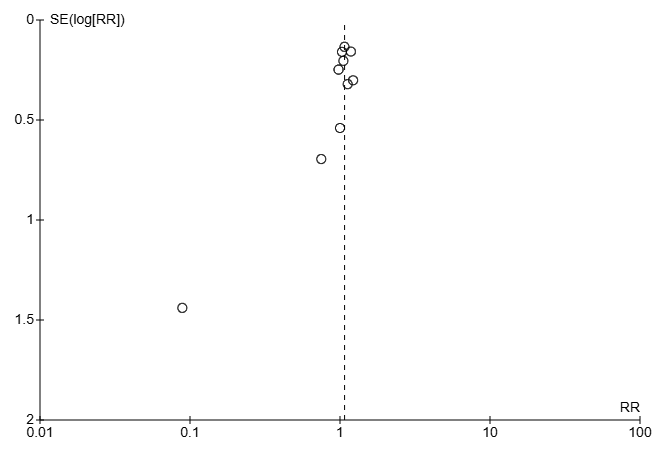


## Fig. S2. Forest plot of subgroup analysis by ARDS etiology for mortality at the longest follow-up


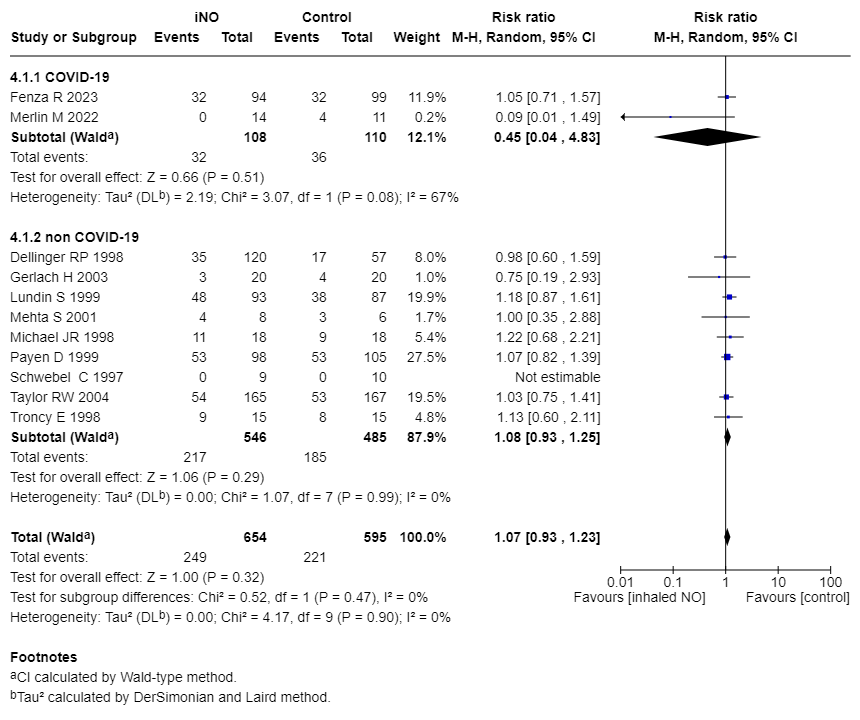


## Fig. S3. Forest plot of subgroup analysis by ARDS severity for mortality at the longest follow-up


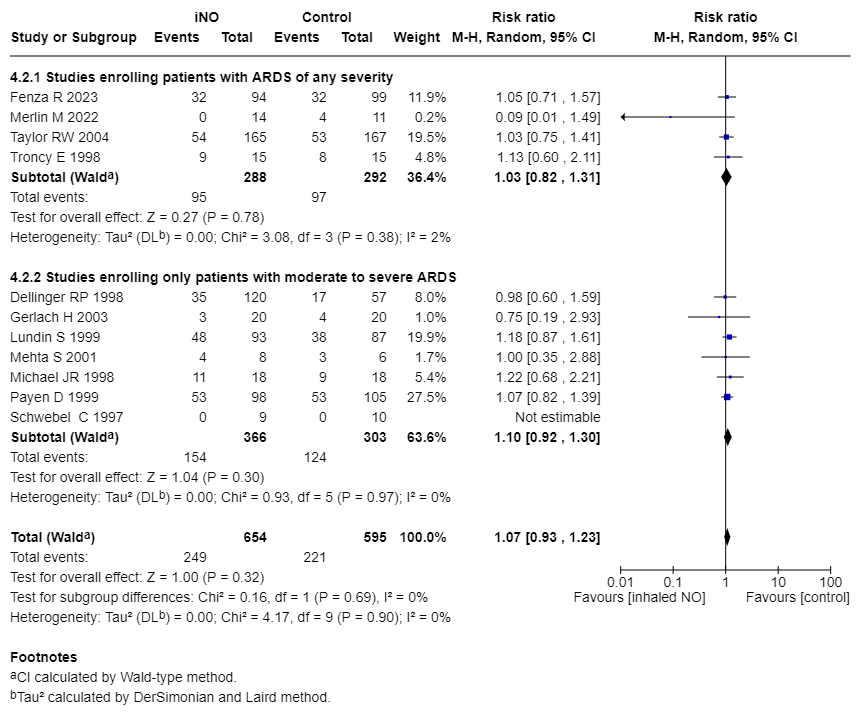


## Fig. S4. Forest plot for mortality at the longest follow-up in studies without high risk of bias


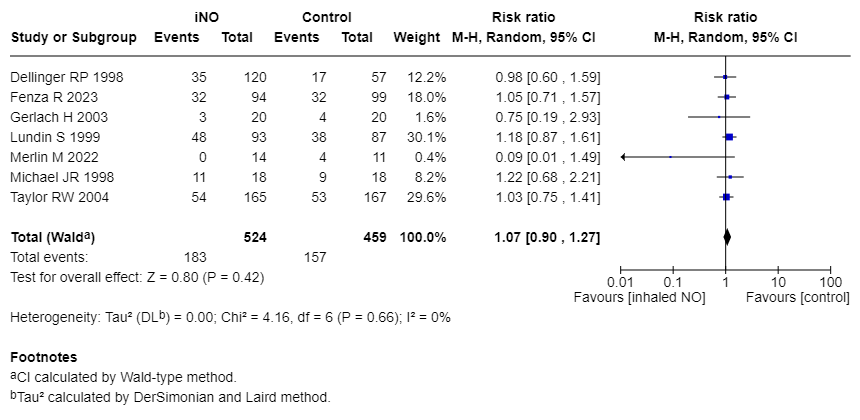


## Table S1. Major exclusions and reasons for exclusion, in order of year of publication

| Author, year | Reason for exclusion |
| --- | --- |
| Rossaint R, 1993 [1] | Full text unavailable |
| Umamaheswara Rao GS, 1996 [2] | Full text unavailable |
| Gallart L, 1998 [3] | Wrong intervention |
| Cuthbertson BH, 1998 [4] | Full text unavailable |
| Cuthbertson BH, 1998 [5] | Full text unavailable |
| Greene JH, 1998 [6] | Full text unavailable |
| Lamb NJ, 1999 [7] | Non-randomized interventional study |
| Venn R, 1999 [8] | Editorial |
| Cuthbertson BH, 2000 [9] | Overlapping study population |
| Gerlach H, 2000 [10] | Congress abstract of an included study |
| Kalassian, K, 2002 [11] | Full text unavailable |
| Wood K, 2002 [12] | Full text unavailable |
| Park KJ, 2003 [13] | Wrong comparator |
| Andalkar L, 2004 [14] | Congress abstract of an included study |
| Angus DC, 2006 [15] | Secondary analysis of an included study |
| Dellinger RP, 2012 [16] | Secondary analysis of an included study |
| Wolak T, 2021 [17] | Protocol article |
| Wall OW, 2021 [18] | Secondary analysis of an included study |
| Tatham KC, 2021 [19] | Wrong intervention |
| Kamenshchikov NO, 2021 [20] | Non-randomized interventional study |
| Strickland B, 2022 [21] | Wrong population |
| Tal A, 2022 [22] | Congress abstract of an excluded study |
| Matthews L, 2022 [23] | Observational study |
| Hagemo JS, 2022 [24] | Protocol article |
| Allam MGIM, 2022 [25] | Wrong comparator |
| Gibson L, 2024 [26] | Secondary analysis of an included study |
| Zheng Z, 2024 [27] | Protocol article |
| Price AD, 2024 [28] | Unavailable mortality data |
| Shetty NS, 2024 [29] | Secondary analysis of an included study |
| Wolak T, 2024 [30] | Wrong population |
| Kamenshchikov NO, 2024 [31] | Wrong comparator |
| Shogenova LV, 2025 [32] | Wrong population |
| Friedrich F, 2025 [33] | Wrong population |
| ChiCTR2500107210 | Clinical trial registration without full-text publication |
| ChiCTR2400085788 | Clinical trial registration without full-text publication |
| ChiCTR2200064651 | Clinical trial registration without full-text publication |
| ChiCTR2000035617 | Clinical trial registration without full-text publication |
| ACTRN12622001411730 | Clinical trial registration without full-text publication |
| ISRCTN54758284 | Clinical trial registration without full-text publication |
| ISRCTN53268296 | Clinical trial registration of an included study [34] |
| ISRCTN16806663 | Clinical trial registration of an included study [35] |
| RBR-8nfx26 | Clinical trial registration of an excluded study [33] |
| EUCTR-001329-30-AT | Clinical trial registration without full-text publication |
| EUCTR-001656-18-NO | Clinical trial registration without full-text publication |
| NCT06852924 | Clinical trial registration without full-text publication |
| NCT04776408 | Clinical trial registration without full-text publication |
| NCT04606407 | Clinical trial registration of an excluded study [30] |
| NCT04421508 | Clinical trial registration without full-text publication |
| NCT04411160 | Clinical trial registration of an excluded study [25] |
| NCT04398290 | Clinical trial registration without full-text publication |
| NCT04397692 | Clinical trial registration without full-text publication |
| NCT04388683 | Clinical trial registration without full-text publication |
| NCT04383002 | Clinical trial registration without full-text publication |
| NCT04306393 | Clinical trial registration of an included study [36] |
| NCT04305457 | Clinical trial registration without full-text publication |
| NCT04290871 | Clinical trial registration without full-text publication |
| NCT00159510 | Clinical trial registration without full-text publication |

## Table S2. Risk of bias assessment of the included studies


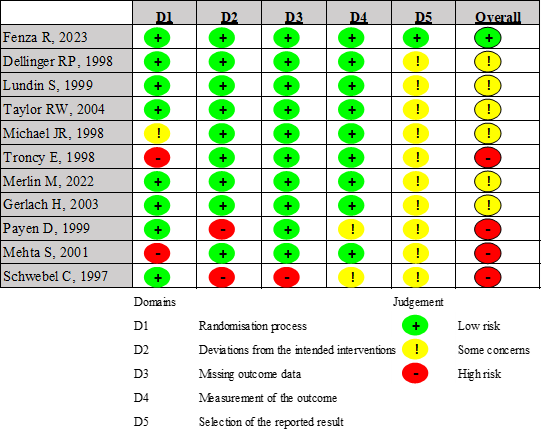


## Table S3. GRADE evaluation

**Author(s):**

**Question:** INO compared to usual care for ARDS

**Setting:**

**Bibliography:**

| **Certainty assessment** | | | | | | | **№ of patients** | | **Effect** | | **Certainty** | **Importance** |
| --- | --- | --- | --- | --- | --- | --- | --- | --- | --- | --- | --- | --- |
| **№ of studies** | **Study design** | **Risk of bias** | **Inconsistency** | **Indirectness** | **Imprecision** | **Other considerations** | **iNO** | **usual care** | **Relative (95% CI)** | **Absolute (95% CI)** |  |  |
| **Mortality at the longest follow-up** | | | | | | | | | | | | |
| 11 | randomized trials | serious^a^ | not serious | serious^b^ | not serious | none | 249/654 (38.1%) | 221/595 (37.1%) | **RR 1.07** (0.93 to 1.23) | **26 more per 1,000** (from 26 fewer to 85 more) | ⨁⨁◯◯ Low^a,b^ | CRITICAL |
| **Acute kidney injury** | | | | | | | | | | | | |
| 5 | randomized trials | serious^c^ | serious^d^ | serious^e^ | serious^f^ | none | 158/597 (26.5%) | 122/541 (22.6%) | **RR 1.34** (0.95 to 1.89) | **77 more per 1,000** (from 11 fewer to 201 more) | ⨁◯◯◯ Very low^c,d,e,f^ | IMPORTANT |
| **Renal replacement therapy** | | | | | | | | | | | | |
| 3 | randomized trials | serious^g^ | not serious | serious^e^ | serious^h^ | none | 89/285 (31.2%) | 58/291 (19.9%) | **RR 1.56** (1.17 to 2.08) | **112 more per 1,000** (from 34 more to 215 more) | ⨁◯◯◯ Very low^e,g,h^ | IMPORTANT |
| **Duration of mechanical ventilation** | | | | | | | | | | | | |
| 4 | randomized trials | serious^a^ | serious^d^ | not serious | serious^h^ | none | 222 | 221 | - | MD **2.24 higher** (3.64 lower to 8.11 higher) | ⨁◯◯◯ Very low^a,d,h^ | IMPORTANT |
| **Length of intensive care unit stay** | | | | | | | | | | | | |
| 3 | randomized trials | not serious | not serious | serious^b^ | serious^h^ | none | 218 | 215 | - | MD **0.2 lower** (2.69 lower to 2.28 higher) | ⨁⨁◯◯ Low^b,h^ | IMPORTANT |
| **Length of hospital stay** | | | | | | | | | | | | |
| 2 | randomized trials | not serious | not serious | serious^b^ | serious^h^ | publication bias strongly suspected^i^ | 198 | 195 | - | MD **0.67 lower** (4.47 lower to 3.13 higher) | ⨁◯◯◯ Very low^b,h,i^ | IMPORTANT |
| **Receipt of extracorporeal membrane oxygenation** | | | | | | | | | | | | |
| 2 | randomized trials | not serious | not serious | not serious | very serious^j^ | publication bias strongly suspected^i^ | 5/114 (4.4%) | 11/119 (9.2%) | **RR 0.45** (0.10 to 2.17) | **51 fewer per 1,000** (from 83 fewer to 108 more) | ⨁◯◯◯ Very low^i,j^ | CRITICAL |
| **Mean pulmonary artery pressure** | | | | | | | | | | | | |
| 3 | randomized trials | serious^a^ | not serious | serious^b^ | serious^h^ | none | 54 | 36 | - | MD **2.48 lower** (5.48 lower to 0.52 higher) | ⨁◯◯◯ Very low^a,b,h^ | IMPORTANT |
| **PaO2/FiO2 ratio** | | | | | | | | | | | | |
| 8 | randomized trials | serious^a^ | not serious | serious^b^ | not serious | none | 309 | 259 | - | MD **15.03 higher** (6.19 higher to 23.86 higher) | ⨁⨁◯◯ Low^a,b^ | IMPORTANT |
| **Methemoglobin concentrations >5%** | | | | | | | | | | | | |
| 10 | randomized trials | serious^a^ | not serious | not serious | very serious^k^ | publication bias strongly suspected^l^ | 4/587 (0.7%) | 3/522 (0.6%) | **RR 0.90** (0.20 to 4.14) | **1 fewer per 1,000** (from 5 fewer to 18 more) | ⨁◯◯◯ Very low^a,k,l^ | IMPORTANT |
| **Nitrogen dioxide concentrations >3ppm** | | | | | | | | | | | | |
| 6 | randomized trials | serious^a^ | not serious | not serious | very serious^k^ | publication bias strongly suspected^l^ | 3/453 (0.7%) | 0/396 (0.0%) | **RR 3.36** (0.18 to 63.89) | **0 fewer per 1,000** (from 0 fewer to 0 fewer) | ⨁◯◯◯ Very low^a,k,l^ | IMPORTANT |
| **Extubation** | | | | | | | | | | | | |
| 1 | randomized trials | serious^m^ | not serious | not serious | very serious^h^ | none | 6/15 (40.0%) | 5/15 (33.3%) | **RR 1.20** (0.47 to 3.09) | **67 more per 1,000** (from 177 fewer to 697 more) | ⨁◯◯◯ Very low^h,m^ | IMPORTANT |

**CI:** confidence interval; **MD:** mean difference; **RR:** risk ratio

**Explanations**

a. Multiple studies had high risk of bias

b. this outcome was assessed at different timepoints among the included studies.

c. one study had a high risk of bias and multiple studies had some concern

d. Statistical heterogeneity is observed (I² >50%)

e. The definition of AKI and the endpoint differ for each study.

f. wide confidence interval likely due to heterogeneity

g. one study had a high risk of bias and one had some concern

h. wide confidence interval likely due to small sample size

i. Funnel-plot assessment is impossible, and selective publication bias cannot be excluded because only two trials reported the outcome.

j. wide confidence interval likely due to small sample size and total events

k. wide confidence interval likely due to total events are too small

l. Some studies did not report the number of events in the control group

m. Only one study with a high risk of bias reported this outcome

## Table S4. Subgroup analyses of secondary outcomes comparing COVID-19 and non-COVID-19

| Outcome | No. of studies | Inhaled nitric oxide | Control | Risk ratio or mean difference (95% CI) | P value | I^2^ | P for interaction |
| --- | --- | --- | --- | --- | --- | --- | --- |
| Acute kidney injury | 5 | 158/597 (27%) | 122/541 (23%) | 1.34 (0.95 to 1.89) | 0.10 | 58% | 0.01 |
| COVID-19 | 1 | 65/94 (69%) | 69/99 (70%) | 0.99 (0.82 to 1.20) | 0.93 | NA |  |
| Non-COVID-19 | 4 | 93/503 (18.5%) | 53/442 (12.0%) | 1.55 (1.14 to 2.10) | 0.005 | 0% |  |
| Renal replacement therapy | 3 | 89/285 (31%) | 58/291 (20%) | 1.56 (1.17 to 2.08) | 0.002 | 0% | > 0.99 |
| COVID-19 | 1 | 33/94 (35%) | 22/99 (22%) | 1.58 (1.00 to 2.50) | 0.05 | NA |  |
| Non-COVID-19 | 2 | 56/191 (29%) | 36/192 (19%) | 1.58 (1.03 to 2.42) | 0.03 | 20% |  |
| Duration of mechanical ventilation, day | 4 |  |  | 2.24 (-3.64 to 8.11) | 0.46 | 82% | 0.60 |
| COVID-19 | 1 |  |  | -0.06 (-8.18 to 8.06) | 0.99 | NA |  |
| Non-COVID-19 | 3 |  |  | 2.88 (-4.55 to 10.31) | 0.45 | 88% |  |
| ICU length of stay, day | 3 |  |  | -0.20 (-2.69 to 2.28) | 0.87 | 0% | 0.17 |
| COVID-19 | 1 |  |  | -2.08 (-5.75 to 1.59) | 0.27 | NA |  |
| Non-COVID-19 | 2 |  |  | 1.38 (-1.99 to 4.76) | 0.42 | 0% |  |
| Receipt of ECMO | 2 | 5/114 (4.4%) | 11/119 (9.2 %) | 0.45 (0.10 to 2.17) | 0.32 | 45% | 0.19 |
| COVID-19 | 1 | 4/94 (4.3%) | 5/99 (5.1%) | 0.84 (0.23 to 3.04) | 0.79 | NA |  |
| Non-COVID-19 | 1 | 1/20 (5.0%) | 6/20 (30%) | 0.17 (0.02 to 1.26) | 0.08 | NA |  |
| PaO2/FiO2 ratio, mmHg | 8 |  |  | 15.03 (6.19 to 23.86) | 0.0009 | 0% | 0.70 |
| COVID-19 | 1 |  |  | 18.70 (-2.18 to 39.58) | 0.08 | NA |  |
| Non-COVID-19 | 7 |  |  | 14.22 (4.48 to 23.97) | 0.004 | 0% |  |
| Methemoglobin concentrations >5% | 10 | 4/587 (0.7%) | 3/522 (0.6%) | 0.90 (0.20 to 4.14) | 0.89 | 0% | NA |
| COVID-19 | 1 | 0/14 (0%) | 0/11 (0%) |  | NA | NA |  |
| Non-COVID-19 | 9 | 4/573 (0.7%) | 3/511 (0.6%) | 0.90 (0.20 to 4.14) | 0.89 | 0% |  |

Abbreviations: CI, confidence interval; ARDS, acute respiratory distress syndrome; COVID-19, coronavirus disease 2019; ICU, intensive care unit ; ECMO, extracorporeal membrane oxygenation; PaO2, arterial oxygen pressure; FiO2, fraction of inspired oxygen; NA, not applicable

## Table S5. Subgroup analyses of secondary outcomes based on ARDS severity

| Outcome | No. of studies | Inhaled nitric oxide | Control | Risk ratio or mean difference (95% CI) | P value | I^2^ | P for interaction |
| --- | --- | --- | --- | --- | --- | --- | --- |
| Acute kidney injury | 5 | 158/597 (27%) | 122/541 (23%) | 1.34 (0.95 to 1.89) | 0.10 | 58% | 0.02 |
| Any severity ARDS | 2 | 77/286 (27%) | 77/292 (26%) | 1.01 (0.84 to 1.21) | 0.91 | 0% |  |
| Moderate to severe ARDS | 3 | 81/311 (26%) | 45/249 (18%) | 1.55 (1.12 to 2.15) | 0.008 | 0% |  |
| Renal replacement therapy | 3 | 89/285 (31%) | 58/291 (20%) | 1.56 (1.17 to 2.08) | 0.002 | 0% | > 0.99 |
| Any severity ARDS | 1 | 33/94 (35%) | 22/99 (22%) | 1.58 (1.00 to 2.50) | 0.05 | NA |  |
| Moderate to severe ARDS | 2 | 56/191 (29%) | 36/192 (19%) | 1.58 (1.03 to 2.42) | 0.03 | 20% |  |
| Duration of mechanical ventilation, day | 4 |  |  | 2.24 (-3.64 to 8.11) | 0.46 | 82% | 0.03 |
| Any severity ARDS | 2 |  |  | -1.72 (-3.47 to 0.03) | 0.05 | 0% |  |
| Moderate to severe ARDS | 2 |  |  | 6.05 (-0.72 to 12.83) | 0.08 | 58% |  |
| ICU length of stay, day | 3 |  |  | -0.20 (-2.69 to 2.28) | 0.87 | 0% | 0.98 |
| Any severity ARDS | 2 |  |  | -0.27 (-3.67 to 3.14) | 0.88 | 46 |  |
| Moderate to severe ARDS | 1 |  |  | -0.70 (-38.27 to 36.87) | 0.97 | NA |  |
| Receipt of ECMO | 2 | 5/114 (4.4%) | 11/119 (9.2 %) | 0.45 (0.10 to 2.17) | 0.32 | 45% | 0.19 |
| Any severity ARDS | 1 | 4/94 (4.3%) | 5/99 (5.1%) | 0.84 (0.23 to 3.04) | 0.79 | NA |  |
| Moderate to severe ARDS | 1 | 1/20 (5.0%) | 6/20 (30%) | 0.17 (0.02 to 1.26) | 0.08 | NA |  |
| PaO2/FiO2 ratio, mmHg | 8 |  |  | 15.03 (6.19 to 23.86) | 0.0009 | 0% | 0.52 |
| Any severity ARDS | 2 |  |  | 20.03 (2.28 to 37.78) | 0.03 | 0% |  |
| Moderate to severe ARDS | 6 |  |  | 13.38 (3.20 to 23.56) | 0.01 | 0% |  |
| Methemoglobin concentrations >5% | 10 | 4/587 (0.7%) | 3/522 (0.6%) | 0.90 (0.20 to 4.14) | 0.89 | 0% | 0.49 |
| Any severity ARDS | 3 | 0/221 (0.0%) | 1/219 (0.5%) | 0.34 (0.01 to 8.17) | 0.50 | NA |  |
| Moderate to severe ARDS | 7 | 4/366 (1.1%) | 2/303 (0.7%) | 1.21 (0.21 to 6.86) | 0.83 | 0% |  |
| Nitrogen dioxide concentrations >3ppm | 6 | 3/453 (0.7%) | 0/396 (0%) | 3.36 (0.18 to 63.89) | 0.42 | NA | NA |
| Any severity ARDS | 2 | 0/207 (0%) | 0/208 (0%) | Not estimable | NA | NA |  |
| Moderate to severe ARDS | 4 | 3/246 (1.2%) | 0/188 (0%) | 3.36 (0.18 to 63.89) | 0.42 | NA |  |

Abbreviations: CI, confidence interval; ARDS, acute respiratory distress syndrome; COVID-19, coronavirus disease 2019; ICU, intensive care unit ; ECMO, extracorporeal membrane oxygenation; PaO2, arterial oxygen pressure; FiO2, fraction of inspired oxygen; NA, not applicable

## Table S6. Sensitivity analysis for secondary outcomes without high risk of bias

| **Outcome** | **No. of studies** | **Inhaled nitric oxide** | **Control** | **Risk ratio/mean difference (95% CI)** | **P value** | **I^2^** |
| --- | --- | --- | --- | --- | --- | --- |
| Acute kidney injury | 4 | 125/499 (25%) | 122/541 (22%) | 1.38 (0.85 to 2.22) | 0.19 | 65% |
| Renal replacement therapy | 2 | 56/187 (30%) | 32/186 (17.2%) | 1.74 (1.19 to 2.55) | 0.004 | 0% |
| Duration of mechanical ventilation, day | 3 |  |  | 4.34 (-1.53 to 10.21) | 0.15 | 56% |
| ICU length of stay, day | 3 |  |  | -0.20 (-2.69 to 2.28) | 0.87 | 0% |
| Hospital length of stay, day | 2 |  |  | -0.67 (-4.47 to 3.13) | 0.73 | 0% |
| Receipt of ECMO | 2 | 5/114 (4.4%) | 11/119  (9.2 %) | 0.45 (0.10 to 2.17) | 0.32 | 45% |
| Mean pulmonary artery pressure, mmHg | 2 |  |  | -3.22 (-7.81 to 1.38) | 0.17 | 65% |
| PaO**_2_**/FiO**_2_** ratio, mmHg | 5 |  |  | 14.17 (4.71 to 23.62) | 0.003 | 0% |
| Methemoglobin concentrations >5% | 6 | 4/457 (0.7%) | 3/386 (0.6%) | 0.90 (0.20 to 4.14) | 0.89 | 0% |
| Nitrogen dioxide concentrations > 3 ppm | 6 | 3/453 (0.7%) | 0/396 (0%) | 3.36 (0.18 to 63.89) | 0.42 | NA |

Abbreviations: CI, confidence interval; ICU, intensive care unit ; ECMO, extracorporeal membrane oxygenation; PaO_2_, partial pressure of arterial oxygen; FiO_2_, fraction of inspired oxygen

## Supplementary references

1.  Rossaint R, Falke KJ, Keitel M, et al. Effects of nitric oxide and prostacyclin on gas exchange and pulmonary artery pressure in ARDS. Eur Acad Anaesthesiol. 1995;10(0):49.

2.   Umamaheswara Rao GS, Gallart L, Law-Koune, et al. Factors influencing cardiorespiratory effects of almitrine and inhaled nitric oxide in 39 patients with ARDS. In: European Society of Anaesthesiologists Annual Congress; June 1–5, 1996; London, UK. Eur J Anaesthesiol. 1996;76(0):114–115.

3. Gallart L, Lu Q, Puybasset L, Umamaheswara Rao GS, Coriat P, Rouby JJ. Intravenous almitrine combined with inhaled nitric oxide for acute respiratory distress syndrome. The NO Almitrine Study Group. Am J Respir Crit Care Med. 1998;158:1770–7. https://doi.org/10.1164/ajrccm.158.6.9804066

4.  Cuthbertson BH, Galley HF, Webster NR. Effect of inhaled nitric oxide therapy on inflammatory response within the lung in ALI. In: Anaesthetic Research Society Meeting; July 9–10, 1998; Dundee, UK. Br J Anaesth. 1998;81(0):645P.

5.  Cuthbertson BH, Galley HF, Webster NR. Effect of inhaled nitric oxide therapy on oxidant stress during ALI. In: Anaesthetic Research Society Meeting; July 9–10, 1998; Dundee, UK. Br J Anaesth. 1998;81(0):645P–646P.

6.  Greene JH, Klinger JR, Levy M, et al. Inhaled nitric oxide (INO) increases oxygen delivery (DO₂) in the acute respiratory distress syndrome (ARDS) when added to positive end-expiratory pressure (PEEP). Am J Respir Crit Care Med. 1998;157(3):A678.

7.  Lamb NJ, Quinlan GJ, Westerman ST, et al. Nitration of proteins in bronchoalveolar lavage fluid from patients with acute respiratory distress syndrome receiving inhaled nitric oxide. Am J Respir Crit Care Med. 1999;160:1031–1034.

8. Venn R. Nitric oxide in ALI. Critical Care. 1999;2:2181. https://doi.org/10.1186/ccf-1999-2181

9. Cuthbertson BH, Galley HF, Webster NR. Effect of inhaled nitric oxide on key mediators of the inflammatory response in patients with acute lung injury. Crit Care Med. 2000;28:1736–41. https://doi.org/10.1097/00003246-200006000-00006

10.  Gerlach H, Busch T, Keh D, et al. Dose response characteristics during long-term inhalation of nitric oxide in ARDS: a prospective randomized study. Am J Respir Crit Care Med. 2000;161(3):A379.

11. Kalassian K, Linde-Zwirble W, Clermont G, et al. The impact of inhaled nitric oxide therapy on long-term survival and quality of life after ARDS. Am J Respir Crit Care Med. 2002;165(8):A220.

12.  Wood K, Linde-Zwirble W, Clermont G, et al. The effect of inhaled nitric oxide on the hospital costs of acute respiratory distress syndrome (ARDS). Am J Respir Crit Care Med. 2002;165(8):A220.

13. Park KJ, Lee YJ, Oh YJ, Lee KS, Sheen SS, Hwang SC. Combined effects of inhaled nitric oxide and a recruitment maneuver in patients with acute respiratory distress syndrome. Yonsei Med J. 2003;44:219–26. https://doi.org/10.3349/ymj.2003.44.2.219

14.  Andalkar L, Spiegler P. Low-dose inhaled nitric oxide in patients with acute lung injury. Clin Pulm Med. 2004;11(4):261–262.

15. Angus DC, Clermont G, Linde-Zwirble WT, Musthafa AA, Dremsizov TT, Lidicker J, et al. Healthcare costs and long-term outcomes after acute respiratory distress syndrome: A phase III trial of inhaled nitric oxide. Crit Care Med. 2006;34:2883–90. https://doi.org/10.1097/01.CCM.0000248727.29055.25

16. Dellinger RP, Trzeciak SW, Criner GJ, Zimmerman JL, Taylor RW, Usansky H, et al. Association between inhaled nitric oxide treatment and long-term pulmonary function in survivors of acute respiratory distress syndrome. Crit Care. 2012;16:R36. https://doi.org/10.1186/cc11215

17. Wolak T, Kalaora R, Hatan M, et al. Inhaled nitric oxide for the treatment of COVID-19 and other viral pneumonias in adults. Am J Respir Crit Care Med. 2021;203(1_MeetingAbstracts):A3849.

18.  Wall OW, Törnberg DT, Hedenstierna MH, et al. Cardiac dysfunction in intubated COVID-19 and the effects of inhaled nitric oxide. Crit Care. 2021;25(0).P062

19. Tatham KC, Ferguson ND, Zhou Q, Hand L, Austin P, Taneja R, et al. Evolution of practice patterns in the management of acute respiratory distress syndrome: A secondary analysis of two successive randomized controlled trials. J Crit Care. 2021;65:274–81. https://doi.org/10.1016/j.jcrc.2021.06.017

20.  Kamenshchikov NO, Kozlov BN, Dish AY, et al. Abstract 11986: A safety study of intermittent versus continuous inhaled NO therapy in spontaneously breathing COVID-19 patients: a randomized controlled trial. Circulation. 2021;144:A11986.

21. Strickland B, Albala L, Coffey EC, Carroll RW, Zapol WM, Ichinose F, et al. Safety and practicality of high dose inhaled nitric oxide in emergency department COVID-19 patients. Am J Emerg Med. 2022;58:5–8. https://doi.org/10.1016/j.ajem.2022.04.052

22. Tal A, Shifer Y, Wolak T, Grossman A, Dicker D. 2190. Characterization of Inhaled Nitric Oxide (iNO) for the treatment of Viral Community Acquired Pneumonia (CAP). Open Forum Infectious Diseases. 2022;9:ofac492.1809. https://doi.org/10.1093/ofid/ofac492.1809

23. Matthews L, Baker L, Ferrari M, Sanchez W, Pappachan J, Grocott MP, et al. Compassionate use of Pulmonary Vasodilators in Acute Severe Hypoxic Respiratory Failure due to COVID-19. J Intensive Care Med. 2022;37:1101–11. https://doi.org/10.1177/08850666221086521

24. Hagemo JS, Skulberg AK, Rehn M, Valberg M, Pesonen M, Heimdal HJ, et al. Inhaled nitric oxide as temporary respiratory stabilization in patients with COVID-19 related respiratory failure (INOCOV): Study protocol for a randomized controlled trial. PLoS One. 2022;17:e0268822. https://doi.org/10.1371/journal.pone.0268822

25. Mostafa Allam MGI, Raddah Alharthi AA, Masfer Alharthi HM. Comparative Study Between Action Vitamin C vs. Action of Nitric Oxide in Prolonged Ventilation in Respiratory Failure Patients Due to ARDS. J Clin Anesthes Res. 2022;3:1–10. https://doi.org/10.52916/jcar224010

26. Gibson L, Di Fenza R, Santiago RDS, Chang M, Berra L. 200: REDUCTION IN RIGHT VENTRICULAR AFTERLOAD WITH NITRIC OXIDE PREDICTS SURVIVAL IN COVID-19 ARDS. Critical Care Medicine. 2024;52:S75. https://doi.org/10.1097/01.ccm.0000998988.00678.7a

27. Zheng Z, Wang L, Wang S, Fan Q, Zhang H, Luo G, et al. Inhaled Nitric Oxide ReDuce postoperatIve pulmoNAry complicaTions in patiEnts with recent COVID-19 infection (INORDINATE): protocol for a randomised controlled trial. BMJ Open. 2024;14:e077572. https://doi.org/10.1136/bmjopen-2023-077572

28. Price AD, Baucom MR, Blakeman TC, Smith M, Gomaa D, Caskey C, et al. Just Say NO: Inhaled Nitric Oxide Effect on Respiratory Parameters Following Traumatic Brain Injury in Humans and a Porcine Model. J Surg Res. 2024;296:497–506. https://doi.org/10.1016/j.jss.2023.12.045

29. Shetty NS, Giammatteo V, Gaonkar M, Li P, Akeju O, Arora G, et al. Differences in the Response to High-Dose Inhaled Nitric Oxide in Self-identified Black and White Individuals: A Post Hoc Analysis of the NOSARSCOVID Randomized Clinical Trial. Am J Respir Crit Care Med. 2024;209:887–90. https://doi.org/10.1164/rccm.202310-1852LE

30. Wolak T, Dicker D, Shifer Y, Grossman A, Rokach A, Shitrit M, et al. A safety evaluation of intermittent high-dose inhaled nitric oxide in viral pneumonia due to COVID-19: a randomised clinical study. Sci Rep. Nature Publishing Group; 2024;14:17201. https://doi.org/10.1038/s41598-024-68055-w

31. Kamenshchikov NO, Safaee Fakhr B, Kravchenko IV, Dish AY, Podoksenov YK, Kozlov BN, et al. Assessment of continuous low-dose and high-dose burst of inhaled nitric oxide in spontaneously breathing COVID-19 patients: A randomized controlled trial. Nitric Oxide. 2024;149:41–8. https://doi.org/10.1016/j.niox.2024.06.003

32. Shogenova LV, Владимировна ШЛ. Efficiency and safety of the integrated use of medical gases thermal heliox, nitric oxide and molecular hydrogen in patients with exacerbation of chronic obstructive pulmonary disease complicated by hypoxemic, hypercapnic respiratory failure and secondary pulmonary arterial hypertension in the post-COVID period. Terapevticheskii arkhiv. 2025;97:242–9. https://doi.org/10.26442/00403660.2025.03.203131

33. Friedrich F, Cypel M, Michaelsen VS, Brum MOA, Ramos F, Marco R, et al. Inhaled nitric oxide for the treatment of COVID-19: an open-label, parallel, randomised controlled trial. ERJ Open Research [Internet]. European Respiratory Society; 2025 [cited 2025 Nov 5];11. https://doi.org/10.1183/23120541.00006-2024

34. Taylor RW, Zimmerman JL, Dellinger RP, Straube RC, Criner GJ, Davis K, et al. Low-dose inhaled nitric oxide in patients with acute lung injury: a randomized controlled trial. JAMA. 2004;291:1603–9. https://doi.org/10.1001/jama.291.13.1603

35. Moni M, Madathil T, Sathyapalan DT, Menon V, Gutjahr G, Edathadathil F, et al. Clinical Efficacy of Inhaled Nitric Oxide in Preventing the Progression of Moderate to Severe COVID-19 and Its Correlation to Viral Clearance: Results of a Pilot Study. Infectious Microbes & Diseases. 2022;4:26. https://doi.org/10.1097/IM9.0000000000000079

36. Di Fenza R, Shetty NS, Gianni S, Parcha V, Giammatteo V, Safaee Fakhr B, et al. High-Dose Inhaled Nitric Oxide in Acute Hypoxemic Respiratory Failure Due to COVID-19: A Multicenter Phase II Trial. Am J Respir Crit Care Med. 2023;208:1293–304. https://doi.org/10.1164/rccm.202304-0637OC
